# CEOT Variants or Entities: Time for a Rethink? A Case Series with Review of the Literature

**DOI:** 10.1007/s12105-020-01200-9

**Published:** 2020-07-08

**Authors:** B. S. M. S. Siriwardena, Paul M. Speight, Christopher D. Franklin, Rasha Abdelkarim, Syed Ali Khurram, Keith D. Hunter

**Affiliations:** 1 Unit of Oral and Maxillofacial Medicine, Pathology and Surgery, School of Clinical Dentistry, University of Sheffield, 19 Claremont Crescent, Sheffield, S10 2TA UK; 2 Department of Oral Pathology, Faculty of Dental Sciences, University of Peradeniya, Peradeniya, Sri Lanka; 3 Oral Pathology and Biology, University of Pretoria, Pretoria, South Africa

**Keywords:** Odontogenic, Tumor, CEOT, Calcifying epithelial odontogenic tumor, Clear cell, Amyloid, *EWSR1*

## Abstract

The first detailed description of calcifying epithelial odontogenic tumor (CEOT) are ascribed to Jens Pindborg, but this tumor was described some years previously. Subsequently, CEOT was included in the 1971 WHO classification of odontogenic tumors and a since then number of variants have been described, which have added confusion to the diagnostic criteria. We aimed to survey the literature on the variants of CEOT, in parallel with a review of our single institution experience of CEOTs. Cases identified were collated, including available clinical, radiological and histological information and then reviewed, taking into account changes in the understanding and classifications of odontogenic tumors since initial diagnosis. We identified 26 cases from 1975 to 2017 for which histological material was available. Of these, only 13 (50%) showed the “classic” histological appearance, whilst two cases were identified as recognized variants. In 11 cases, other diagnoses or a differential diagnosis were preferred, with no agreed diagnosis in four of these. The proliferation fraction (Ki67) in the 10 cases tested was 2.1% ± 0.18. These findings illustrate the diagnostic challenges in this group of tumors and highlight the gaps in knowledge. Techniques, such as *EWSR1* gene cytogenetic analysis, may be helpful in cases with clear cells. However, in other areas of controversy, including the non-calcifying and Langerhans cell rich variants, further investigation, perhaps utilizing sequencing technologies may be needed to refine the classification. Owing to the relative rarity of these lesions it would be beneficial if future work could be pursued as an international collaboration.

## Introduction and Review of the Literature

Jens Pindborg described the calcifying epithelial odontogenic tumor (CEOT), a rare epithelial odontogenic tumor, in detail in 1958 [1]. Many authorities suggest, however, that the first description was by Thoma and Goldman ten years previously, who termed it adenoid-type adamantoblastoma [2], although earlier descriptions do exist [3]. Various synonyms have been used to describe this lesion, such as adamantoblastoma [4], ameloblastoma of unusual type with calcification [5], malignant odontoma [6], and cystic complex odontoma [7]. In 1963, the term ‘Pindborg tumor’ was first used by Shafer and this is a well-recognized eponym for this neoplasm [8]. Twenty years after the original CEOT description, Pindborg and Franklin reviewed 113 cases reported in the literature [9].

Since the original descriptions, the number of cases has continued to increase and, to date, more than 362 cases have been reported [10]. According to this recent review of published cases, there was an almost equal distribution among males and females and the peak age of occurrence of central lesions was in the 3rd and 4th decades, similar to that presented in our recent series of odontogenic tumors [11]. The majority occurred in the body of the mandible, but some were large lesions, extending widely antero-posteriorly and involving the ramus [10, 11]. Most presentations are intraosseous but in 1966, Pindborg described an extra-osseous/peripheral CEOT [12].

Radiologically, CEOTs vary from small, unilocular radiolucent lesions to extensive multilocular, mixed radio-dense lesions often associated with an impacted tooth (in 61% of central cases [10]). Some authors have considered the presence of radio-opaque flecks in the pericoronal tissues of an impacted tooth (as originally described by Pindborg) as characteristic for CEOT [13]. Half of the central lesions show evidence of cortical bone perforation whilst 40% of peripheral CEOTs have subjacent bone erosion [10]. On Computed tomography (CT) scans, there is diffuse high attenuation, suggesting calcification and/or ossification. On magnetic resonance imaging (MRI), CEOT is a hypointense tumor on T1-weighted images and a mixed hyper intense tumor on T2-weighted images [14]. CT scans and 3D reconstructions may be useful in delineating the extent of the lesion, which is essential for surgical treatment planning [15]. Whilst CEOT is considered a benign epithelial neoplasm, evidence of clinically aggressive behavior, malignant transformation with multiple recurrences and cases with metastasis have been reported [10, 16].

**Fig. 1:**
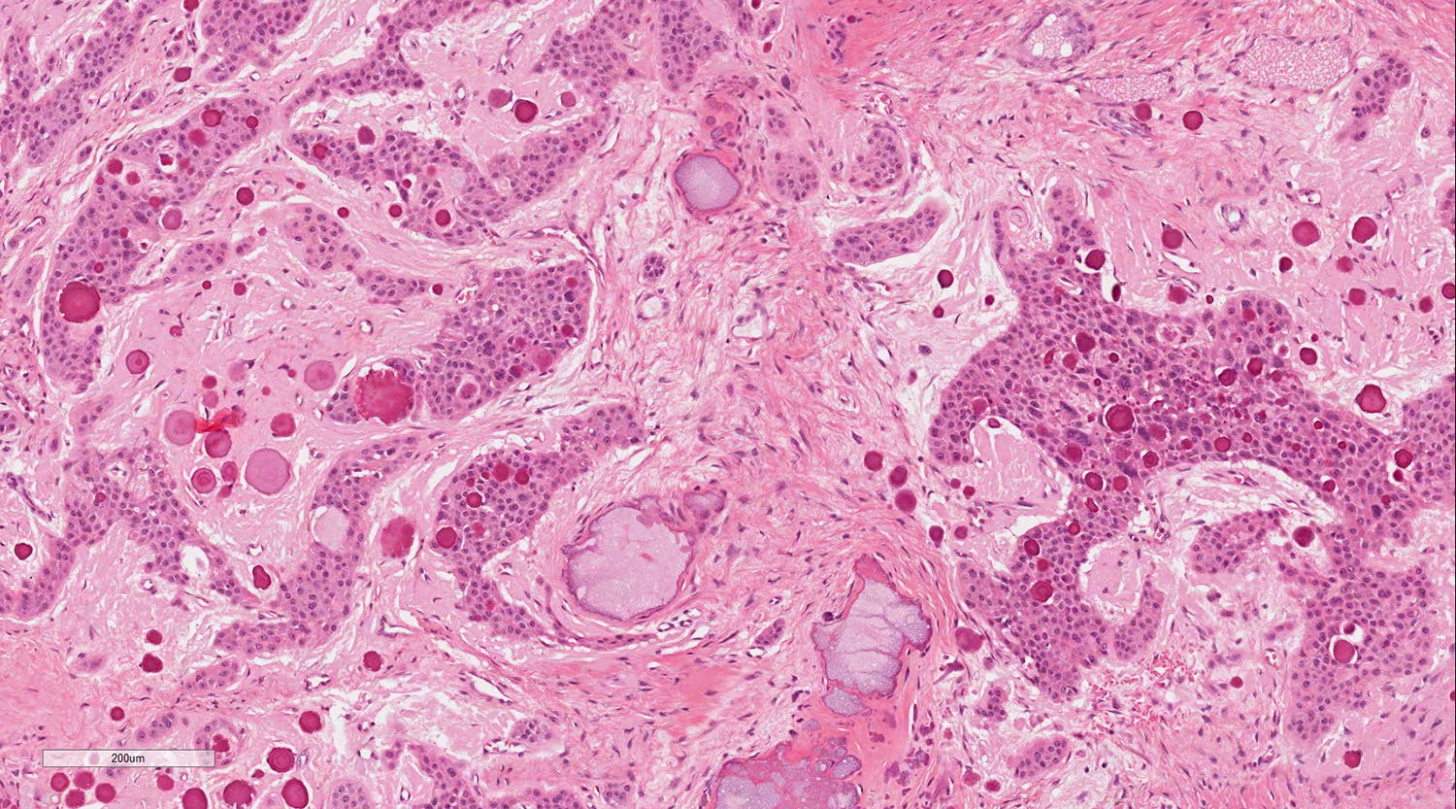
Photomicrograph illustrating the histological features described the original publication by Pindborg [1]

**Fig. 2:**
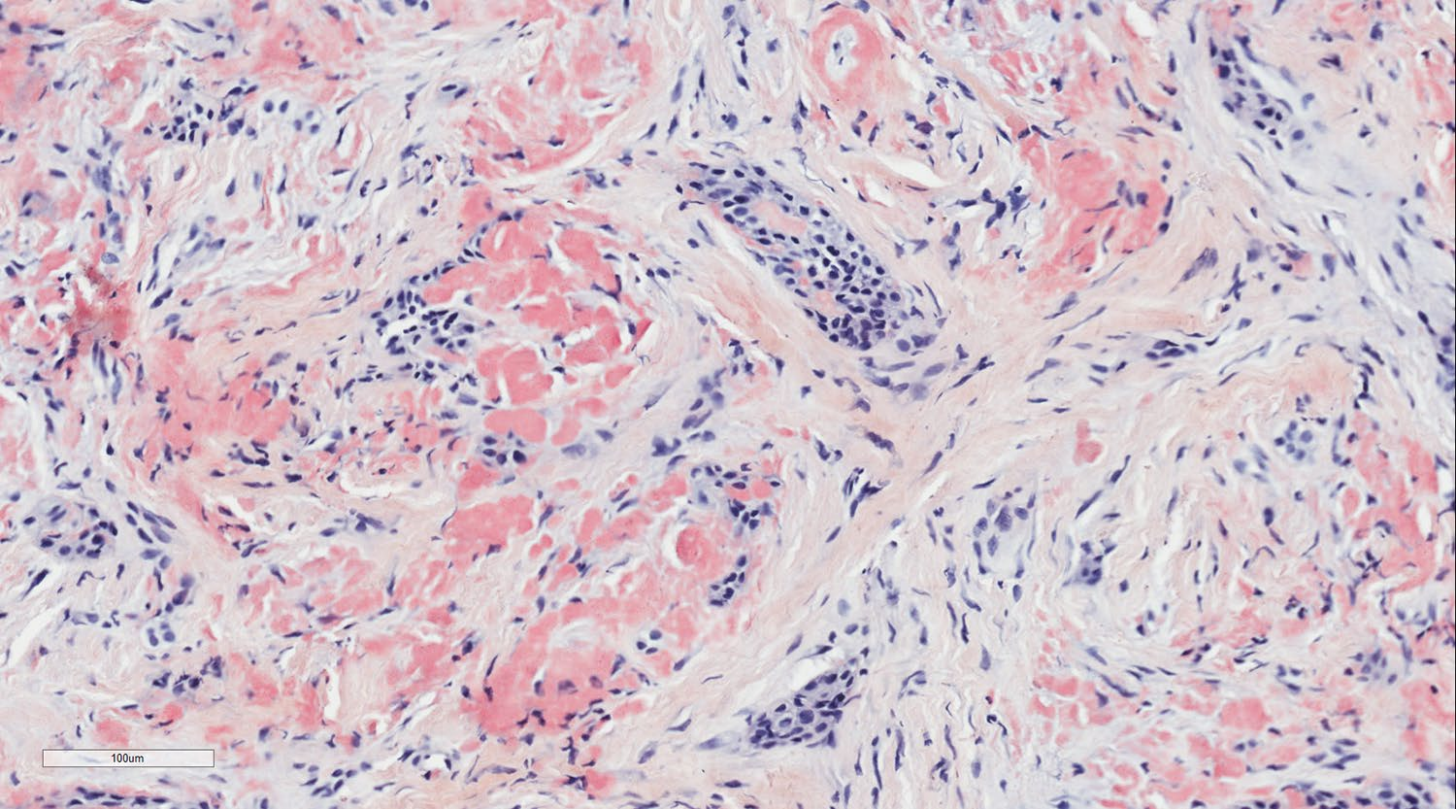
Photomicrograph of the characteristic appearance of CEOT amyloid, as stained by Congo Red (**a**)

The histological hallmarks of the “classic” CEOT are sheets of polyhedral epithelial cells with distinct cell borders, prominent intercellular bridges, nuclear pleomorphism, and few mitoses (Fig. 1) [1, 9, 12]. Also common are concentric calcifications (Liesegang rings) and the presence of deposits of amorphous ‘amyloid-like’ eosinophilic material which stains with Congo Red (Fig. 2) and demonstrates apple-green birefringence on polarization. This material is largely PAS negative prior to calcification [9].

It has been suggested that CEOTs originate from remnants of the dental lamina [17] or stratum intermedium [18]. Two cell types have been demonstrated by electron microscopy: polyhedral epithelial cells and myoepithelial-like cells containing electron-dense tonofilament bundles, electron-dense bodies, and fine lamina dense filaments [19]. Immunohistochemically, the polyhedral cells of CEOT express laminins 1 and 5, cytokeratins, fibronectin and vimentin [20]. High levels of alkaline phosphatase and ATPase localization to the cell membrane are significant findings [21]. The amyloid material has been shown to contain a number of ameloblast associated proteins, most consistently Odontogenic Ameloblast-Associated Protein (ODAM) [22].

Apart from the classic features, a number of CEOT variants have been reported, with various proportions of clear cells, Langerhans cells and some cases without calcification. Furthermore, hybrid tumors with adenomatoid odontogenic tumor or ameloblastoma [10, 23, 24], and cystic/microcystic variants have been reported [25, 26]. Ai-Ru et al. proposed a sub-classification comprising four histological patterns, indicating that some tumors might show a cribriform appearance without clear cell borders; others may contain multinucleated giant cells or cells with abundant eosinophilic cytoplasm or clear/vacuolated cells with centrally placed nuclei [27]. However, this sub-classification was based on only nine cases and has not been widely adopted or otherwise assessed in a larger study population.

In this case series, we aimed to review all of our diagnoses of CEOT in the diagnostic archive (either definitive or in differential diagnosis) and review them in light of the three WHO classifications published during this time (1991, 2005 and 2017) and the current literature on this entity.

## Materials and Methods

The diagnostic database of the department of Oral and Maxillofacial Pathology, Charles Clifford Dental Hospital/School of Clinical Dentistry, University of Sheffield, was searched for cases either with the diagnostic code of CEOT (as a definitive diagnosis) or by keyword search where CEOT was raised as a differential diagnosis in more challenging cases from 1975 to 2017. Clinical information including age, gender and location of the tumor were recorded, and plain film radiology was reviewed where available. Very limited clinical follow-up data was available, and none of the cases for which this was available recurred.

Given the passage of time since the original diagnoses in the series (a span of 42 years: and three intervening WHO classifications), the original slides were re-evaluated using contemporary diagnostic criteria, with attention to the 2017 WHO classification of odontogenic lesions [28]. Hematoxylin and Eosin and Congo Red stained sections of the selected cases from the database were re-evaluated by 3 experienced OMF Pathologists (PMS, KDH and SAK), and consensus diagnoses recorded. Cases with multiple biopsies (incisional and resection) were considered as single cases.

Immunohistochemical analysis of the expression of Ki67 (Rabbit polyclonal Abcam ab16667 at 1:50; to assess the proliferation fraction) and Amelogenin/AMELX (Rabbit monoclonal, Abcam ab129418 at 1:150; to assess ameloblastic differentiation) was conducted on 10 and 8 cases respectively, where sufficient formalin fixed paraffin embedded (FFPE) material remained. Slides were dewaxed and rehydrated before quenching of endogenous peroxidase using H_2_O_2_. Heat-induced epitope retrieval in 0.01 M sodium citrate was undertaken before blocking with normal serum. After primary antibody incubation, biotinylated secondary antibodies were used and specific staining demonstrated using the Vector Nova Red kit (Vector Laboratories Inc, Burlingame, CA, USA). Ki67 was assessed as % of cells positive and AMELX expression was assessed using a modified quickscore method [29], with a maximum possible score of 24.

## Results

Thirty two cases had been coded as CEOT in the diagnostic database from 1975 to 2017. Histological slides (H&E and Congo Red) were available for 26 cases (Table 1). In one additional case, whilst a differential diagnosis of CEOT was suggested in the incisional biopsy, the resection showed an unequivocally malignant odontogenic tumor. This case was excluded. A variety of other histochemical (largely PAS) and immunohistochemical stains were available in some cases, conducted as part of the original diagnostic work-up. Of the 26 cases, 18 were referral/consult cases, so the FFPE blocks were not available for further analysis. In 15 cases, a definitive diagnosis of CEOT had been made, whilst in the remaining 11, it was part of a differential diagnosis.

**Table 1:** Demographic and histological data of the cohort of 26 CEOTs

Case no	Year of Dx	Age	Sex	Site	Central/peripheral
1	1975	32	Male	Not known	Central
2	1978	38	Female	Not known	Central
3	1980	50	Female	Not known	Peripheral
4	1982	38	Male	Mid Mandible	Central
5	1988	25	Male	Mid to post mandible	Peripheral
6	1992	23	Male	Ant to mid maxilla	Peripheral
7	1993	39	Female	Mid to post mandible	Central
8	1993	31	Female	Ant to mid mandible	Central
9	1997	44	Male	Mid to post mandible	Central
10	1998	52	Male	Mid mandible	Central
11	1999	49	Female	Mid maxilla	Central
12	2003	32	Female	Ant mandible	Peripheral
13	2004	69	Female	Mid maxilla	Central
14	2004	25	Male	Maxillary antrum	Central
15	2007	48	Female	Post mandible	Central
16	2008	53	Male	Maxillary antrum	Central
17	2009	30	Male	Post maxilla	Central
18	2010	47	Male	Mid to post mandible	Peripheral
19	2010	27	Female	Ant to mid mandible	Peripheral
20	2011	46	Male	Mid to post maxilla	Central
21	2011	49	Male	Mid to post mandible	Central
22	2012	74	Female	Ramus of mandible	Central
23	2013	52	Male	Mid Mandible	Central
24	2015	32	Female	Ant maxilla	Peripheral
25	2015	55	Female	Maxillary antrum	Central
26	2016	34	Female	Maxillary antrum	Peripheral

*Dx* diagnosis

The age range was 23–74 years with a mean age of 42 ± 2.6 (Table 1). There was an equal gender distribution. 62% occurred in the mandible and, of the mandibular tumors, the majority were in the posterior mandible (54%). Of those in the maxilla, 3/10 (30%) involved the maxillary sinus. The majority of CEOTs were intraosseous (18/26; 69%), whilst 8 were peripheral lesions (31%). Association with unerupted teeth was not consistently recorded.

Histologically, a variety of appearances were seen and many cases met the criteria for diagnosis originally described by Pindborg (13/26; 50%), but a number of other histological appearances were also observed. Clear cell clusters (of varying extent) were observed in 46% (12/26), more commonly in peripheral tumors (6/8; 75%). Out of the total sample, 10 cases had no identifiable calcifications (Table 2). Three of the cases (7, 24 and 26) contained dentin-like material (dentinoid).

**Table 2:** Histological features of the cohort of 26 CEOTs

Case no	Epithelium description	Distinct cellular outline	Prominent intercellular bridges	Eosinophilic cytoplasm	Nuclear/cellular pleomorphism	Mitotic figures	Calcifications/ Liesegang rings	Amyloid	Clear cells	Original diagnosis	Review consensus diagnosis	IHC
1	Nests	Y	Y	Y	Y	N	Y	Y	N	Typical CEOT	CEOT	
2	Nests	Y	N	Y	Y	N	N	Y	N	Typical CEOT	CEOT	ki67 6%
3	Small nests and thin strands	Y	N	Y	Y	N	N	Y	Y (focal)	Unusual, maybe CEOT or OD hamartoma	CEOT vs OdF	
4	Nests	Y	Y	Y	Y	N	Y	Y	N	Typical CEOT	CEOT	
5	Small nests and thin strands	N	N	Y	Y	N	Y (focal)	N	Y (focal)	Unusual, CEOT (preferred) vs OdF	CEOT vs CCOC	ki67 5%
6	Small nests and thin strands	Y	N	Y	Y	N	N	Y	Y (focal)	CEOT	CEOT vs OdF	
7	Sheets and thin strands	Y	N	Y	Y	N	Y*	N	Y	CEOT, clear cell variant	No consensus	ki67 < 1%
8	Sheets and small nests	Y	Y	Y	Y	N	Y	Y	N	Typical CEOT	CEOT	ki67 2%
9	Sheets and thin strands	N	N	Y	Y	N	N	Y	N	Unusual OT, CEOT vs OF	No consensus	
10	Sheets and thin strands	Y	N	Y	Y	N	Y	Y	Y (focal)	CEOT	CEOT, clear cell variant	ki67 < 1%
11	Small nests and thin strands	Y	N	Y	Y	N	N	Y	N	CEOT	CEOT vs OdF/SOC	ki67 2%
12	Small nests and thin strands	Y	Y	N	Y	N	Y	Y	Y	CEOT	CEOT	
13	Small nests	Y	N	Y	Y	N	Y (min)	Y	Y	CEOT	CEOT	ki67 < 1%
14	Small nests	Y	Y	Y	Y	N	Y	Y	Y (focal)	CEOT, maybe arising from dentigerous cyst	CEOT	ki67 1%
15	Small nests	Y	Y	Y	Y	N	Y	Y	N	Typical CEOT	CEOT	
16	Sheets and thin strands	Y	N	Y	Y	N	N	N	Y	Unusual, maybe CEOT variant	Ameloblastoma with clear cells	
17	Small nests and thin strands	Y	Few	?	Y	N	N	N	N	Unusual, perhaps non-calcifying CEOT	No consensus	
18	Small nests and thin strands	Y	In some areas	Y	Y	N	Y	Equiv	Y (most)	Clear cell CEOT	CEOT, clear cell variant	
19	Small nests and thin strands	Y	Y	Y	Y	N	N	Y	N	CEOT	CEOT	
20	Small nests	Y (few)	N	Y	Y	N	Y (few)	Y	Y (focal)	CEOT	CEOT vs OdF	
21	Sheets	Y	N (few)	Y	Y	N	Y	Y	Y	Unusual, perhaps CEOT	No consensus	ki67 < 1%
22	Sheets	Y	N (few)	Y	Y	N	Y	Y	N	CEOT	CEOT	
23	Small nests and thin strands	Y	N	Y	Y	N	N	Y	N	CEOT	CEOT	
24	Sheets and thin strands	N	N	Y	Y	N	Y* (few)	Equiv	n	CEOT	CEOT vs OC with dentinoid	
25	Small nests and thin strands	Y	N	Y	Y	N	Y	Y	N	CEOT	CEOT	
26	Small nests and thin strands	Y	N	Y	Y	N	Y*	Y	Y (most)	OT, perhaps CEOT	CEOT, Clear Cell variant vs OC with dentinoid	Ki67 < 1%

*OT* odontogenic tumor, *OdF* odontogenic fibroma, *OC* odontogenic carcinoma, *CCOC* clear cell odontogenic carcinoma, *SOC* sclerosing odontogenic carcinoma, *equiv* equivocal

*Calcifications assessed as “dentinoid”

The relationship of the review diagnoses to the original diagnoses is presented in Table 2. Of the 26 cases, 14 were confirmed as CEOT (12 “classic” CEOT, and 2 of the clear cell variant of CEOT). In 6 cases, CEOT was part of a differential diagnosis, which variably included central odontogenic fibroma, clear cell odontogenic carcinoma (CCOC), sclerosing odontogenic carcinoma and odontogenic carcinoma with dentinoid. In two cases, other diagnoses were favored (one clear cell odontogenic carcinoma, and one ameloblastoma with clear cells), and four were odontogenic tumors which were difficult to classify with no consensus achieved.

**Fig. 3:**
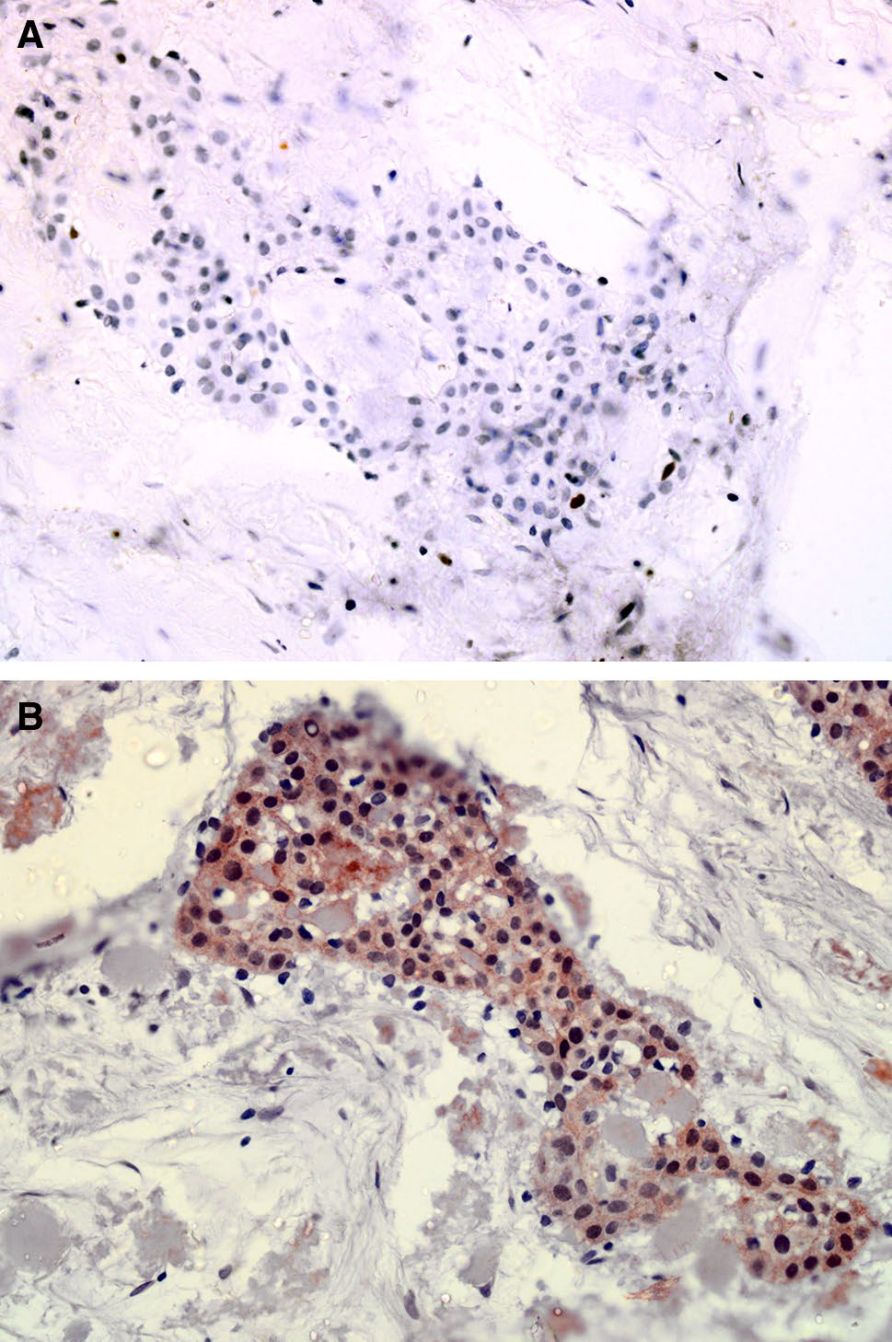
Photomicrograph of Ki67 (**a**) and AMELX expression (**b**) in a selected CEOT case from the cohort

Immunohistochemistry for Ki67 expression was available for 10 of the cases with a mean of 2.1% of positive cells (SEM = 0.18; range 1–6%; Fig. 3a). This reinforces the concept that despite frequent nuclear and pleomorphism, the proliferation rate is low. There was no discernible pattern of ki67 expression with regard to histological subtype, nor in those cases where a malignant diagnosis was considered. The lowest (1%) and highest (6%) Ki67 expression were both found in “classic” subtypes. AMELX (amelogenin) was expressed in the epithelium in all 8 cases tested, with the histoscore varying between 5 and 18 (Fig. 3b), indicating that this may be of use, similar to ODAM, in demonstrating ameloblastic differentiation in the epithelial cells.

## Discussion

A summary of the main histological variants of CEOT, which have been described in the literature, is presented in Table 3 and a summary of the histochemical and immunohistochemical staining characteristics of these different cell types is presented in Table 4. In addition to these main variants, others, such as melanin-containing lesions have also been described [24, 30]. The reported variation in clinical outcomes may represent a spectrum of biological behavior in CEOT, but conversely may merely represent a group of heterogeneous entities which have, for various reasons discussed below, been classified together as “variants” of CEOT, which are briefly reviewed below.

**Table 3:** A summary of reported cases and case series of variants of CEOT

Authors	Age/sex	Location	Radiographic features	Histopathological findings	C/P
*CEOT cystic variant*					
Gopalakrishnan et al. [26]	15M	Left posterior maxilla	Unilocular radiolucency with radiopacities	Cyst lining varying from NKSSE to thickened epithelium with characteristics of CEOT	C
Channappa et al. [48]	30M	Left posterior maxilla	Unilocular radiolucency with calcifications in association with impacted tooth #13	Cyst lined by odontogenic epithelium, majority with uniform thickness, with classic features of CEOT	C
Urias Barreras et al. [49]	31M	Left Posterior mandible	Unilocular radiopaque/lucent area	Lining of odontogenic epithelium with necrosis, featuring clear cells (PASD positive and osteodentin	C
Dantas et al. [50]	22M	Right posterior mandible	Unilocular, mixed radiodensity lesion, root resorption	Microcystic lined by typical CEOT with abundant clear cells	C
Sánchez-Romero et al. [25]	42F	Right posterior mandible	Well-defined mixed radiodense lesion in relation to an un-erupted third molar	Microcystic compartments of varying size and occasional clear cells with classic features of CEOT	C
*CEOT clear cell variant*					
Abrams and Howell [31]	50M	Posterior mandible	Unilocular mixed radiodense/radiolucent	Prominent clear cells with classic features of CEOT	C
Anderson et al. [37]	68F	Left mandible molar area	Unilocular radiolucent/radiopacity	Prominent clear cells with classic features of CEOT	C
Oikarinen et al. [59]	36F	Mandible Left molar to right premolar	Multiloculated radiolucent with radiopaque central region	Prominent clear cells with classic features of CEOT. Amyloid diagnosed under electron microscopy	C
Yamaguchi et al. [60]	36M	Right mandible from anterior to premolar region	Unilocular radiolucency	Prominent clear cells with classic features of CEOT. PAS positive granules in clear cells	C
Ai-Ru et al. [27]	64F	Anterior mandible	Not recorded	Prominent clear cells with classic features of CEOT	C
Asano et al. [43]	44F	Right maxilla	Unilocular, radiolucent area with root resorption	Islands that frequently contained clear cells with typical features of CEOT	C
Schmidt-Westhausen et al. [36]	38M	Right premolar to left incisor region in mandible	Radiolucency with diffuse radiopacities in part of the lesion	Central necrosis of large epithelial islands and clusters of clear cells	C
Hicks et al. [61]	59F	Right posterior mandible	Unilocular mixed radiolucency and radiopacity	Prominent clear cells with classic features of CEOT	C
Kumamoto et al. [62]	14F	Right maxillary 3rd molar region	Unilocular radiolucency impacted upper right 3rd molar	Prominent clear cells, few mitotic figures and typical features of CEOT	C
Anavi et al. [33]	27M	Left mandibular canine and first premolar	Unilocular well-circumscribed radiolucency	Sheets of clear cells, amyloid and few small oval calcifications	C
Germanier et al. [63]	44F	Right angle of the mandible enclosing the 3rd molar	Multiloculated radiolucency with calcifications	Clear cells in some places and with typical CEOT	C
Mohtasham et al. [64]	18M	Right anterior maxilla	Radiolucency with calcification	Scattered clear cells with typical features of CEOT	C
Rangel et al. [32]	65M	Right mandible between lateral incisor and canine	Unilocular radiolucency with radio-opacities	Significant portion of cells are clear and other areas with typical features of CEOT	C
Sahni et al. [65]	52M	Right maxilla	Mixed radiodensity lesion	Areas of clear cells within epithelial islands and with typical features of CEOT	C
Chen et al. [66]	59F	Posterior mandible/ ramus	Unilocular radiolucency	Nests of clear cells in a pseudoglandular pattern. Other areas with typical features of CEOT	C
Turatti et al [67]	25F	Left mandible	Unilocular radiolucency with root displacement	Sheets and nests of clear cells with areas of calcifications and amyloid	C
Rydin et al. [68]	40F	Left mandible	Unilocular radiolucency with scattered calcifications	Central portion of the tumor composed of clear cells and periphery with typical CEOT	C
Chatterjee et al. [69]	73F	Left maxillary molar region	A large mixed radiodense/RL area spearing maxillary antrum	Typical CEOT with Clear cells. PAS positive	C
Sabir et al. [70]	63F	Angle of the mandible	Radiolucent lesion in ramus distal to 3rd molar	Almost all islands are clear cells amyloid in between	C
Júnior et al. [71]	42M	Mandibular symphysial region	Unilocular radiolucency with patchy radio density	Most clusters with clear cells and abundant small calcifications and amyloid	C
Wertheimer et al. [35]	20M	Right maxillary gingiva	Premolar region cup-shaped area	Typical areas of CEOT with some areas with clear cells	P
Ai-Ru et al. [27]	32F 47F	mandibular gingiva	No signs of bone involvement	Typical areas of CEOT with some areas with clusters of clear cells	P
Houston and Fowler [72]	27M	Gingiva of right posterior mandible	Underlying bone was normal	Prominent clear cells with classic features of CEOT	P
Orsini et al. [73]	32M	Maxillary gingiva	NA	Typical areas of CEOT with some areas with clusters of clear cells	P
Mesquita et al. [74]	48F	Right maxilla, canine region	NA	Polyhedral and clear epithelial cells associated with amyloid-like deposition	P
Anavi et al. [33]	27M	Left mandible	Alveolar crest resorption	Sheets of clear cells, focal mild atypia with amyloid in between cells and clusters	P
de Oliveira et al. [75]	43F	Lesion 1: Left mandible Lesion 2: Left maxilla	Superficial cupping in canine area	some clusters are composed with clear cells with typical features of CEOT	P
Habibi et al. [76]	70F	Left maxilla	Normal underlying alveolar bone	Typical areas of CEOT with some areas with clusters of clear cells	P
Gadodia et al. [77]	18M	Left mandible	Alveolar crest resorption	Scattered clear cells with classic features of CEOT	P
*CEOT Non-calcified with Langerhans cells*					
Asano et al. [43]	44F	Right maxilla	Unilocular radiolucency	Less cellular, clear cells within polyhedral cell clusters. Birbeck granules seen. No calcification	C
Takata et al. [44]	58M	Left maxillary canine premolar region	Unilocular radiolucency	Scattered small islands of epithelial cells. Within islands many spherical bodies seen. Amyloid present. S-100 positive. Birbeck granules identified	C
Wang et al. [78]	52F	Right maxilla, central incisor canine region	Unilocular radiolucency	Small nests of polyhedral cells and amyloid deposition. Clear cells present. CD1a positive cells are frequent. No calcification	C
Wang et al. [79]	38M 39F	Right mandible Left maxilla	Unilocular radiolucency with patchy radiopacities	Small nests and cords of epithelial cells. Few clear cells. Amyloid present. CD1a + , Birbeck granules identified. No calcification	C P
Afroz et al. [40]	20F	Right maxilla, lateral incisor area	Normal underlying alveolar bone	Scattered small islands of polygonal cells and occasional clear cells. Amyloid present. No calcifications. Clear cells confirmed as Langerhans cells (S100)	P
Chen et al. [45]	40F 58M	Maxilla	Unilocular radiolucency with root resorption Multilocular radiolucency with root resorption	Small nests and cords of epithelial islands with some clear cells. Amyloid present, CD1a + , langerin + , No calcification	Both C
Tseng et al. [80]	24M	Left maxilla, canine premolar area	Unilocular radiolucency with root resorption in canine and premolar	Strands and island of epithelial cells and some clear cells. Scant amyloid, CD1a + , No calcification	C
Santosh et al. [81]	44M	Left anterior maxilla	Large unilocular radiolucency	Bland epithelial islands with admixed amyloid. CD1a + cells. No calcification was present	C
*Combined epithelial odontogenic tumor. CEOT /AOT*					
Damm et al. [18]	18M 15F	Mandible	Unilocular predominantly radiolucent, one case with radiopacities	A cystic tumor lined with areas of typical AOT. And some CEOT-like areas	C
Bingham et al. [82]	14F	Right mandible	Unilocular radiolucent lesion related to impacted first premolar tooth	Cystic tumor with multiple intraluminal nodules. Some typical AOT and others are CEOT. Amyloid positive. Calcifications noted	C
Takeda and Kudo [83]	17F	Right maxilla between incisors	Unilocular radiolucent lesion with flakes of radio densities	Encapsulated solid tumor with areas of typical AOT and CEOT. Amyloid positive	C
Siar and Ng [51]	13–28 2M, 3F	3 in maxilla, 2 in mandible	Radiolucent lesion	Thick walled cystic tumor lined with areas of typical AOT and variable amounts of CEOT-like areas	All C
Ledesma et al. [84]	10–21 10F, 2M	9 in maxilla (most canine region), 2 mandible	Radiolucent lesion most related to impacted canine tooth. Some have radiopacities	Typical AOT areas with CEOT-like areas of variable sizes	11 C 1 P
Miyake et al. [85]	16F	Left maxilla, canine region	Radiolucent lesion related to impacted canine tooth	Encapsulated solid tumor composed with areas of typical AOT and CEOT. Amyloid positive	C
Rosa et al. [86]	17	Anterior mandible	Unilocular radiolucent lesion with radio-opacity centrally	A cystic tumor with solid mural nodules with typical AOT and CEOT areas. Amyloid positive	C

*C* Central, *P* Peripheral

**Table 4:** Histochemical and immunohistochemical stains in CEOT

	Epithelial cells	Amyloid- like material	Calcification	Clear cells	Langerhans cell	Stromal cells
Histochemical stains						
Congo red		✔				
Thioflavin T		✔				
PAS		✔_a_				
Tryptophan		✔				
IHC stains						
Pan-cytokeratin		✔		✔		
Cytokeratin cocktail		✔				
EGFR	✔					
p63	✔			✔		
CK7	✔			✔		
CK14	✔			✔		
CK8	✔			✔		
CK13	✔			✔		
CK19	✔			✔		
Vimentin						✔
Ameloblast-associated protein	✔	✔	✔			
Amelotin	✔					
Ameloblastin	✔					
Amelogenin		✔	✔			
S100 protein					✔	
CD1a					✔	
Langerin					✔	
Enamelin		✔				
Syndecan-1 (CD138)	✔	✔	✔			
E-Cadherin	✔					
Amyloid A	✔	✔	✔			

The information has been gathered from references [9, 20–22, 35, 67, 87, 42]

^a^If not calcified

### Clear Cell Variant

In 1967, Abrams and Howell described the first case of a CEOT with a clear cell component [31]. Many case reports and series have followed, some of which are summarized in Table 3. Most of the clear cell CEOTs are intraosseous lesions and are most commonly found in the mandible [10]. The mean age is 44 years, which is 8 years older than for conventional CEOT. Unlike conventional CEOT, there is a female predilection and an association with unerupted teeth was found in only six out of the 24 patients, compared with nearly 50% of the conventional CEOTs. It has been suggested that clear cell CEOTs are clinically more aggressive as they tend to perforate the cortex and recur more frequently than other CEOT variants [32–34].

In almost all the reported cases, there were areas with histological features of conventional CEOT including polyhedral sheets of epithelial cells with prominent intercellular bridges, amyloid-like material and calcifications. The clear cells contain PAS positive material which is diastase labile, consistent with glycogen, and does not stain with Alcian Blue [35]. This finding is consistent with suggestions that the clear cells form by epithelial cell degradation [36, 37]. Although the presence of typical areas of conventional CEOT, with minor cellular atypia and absence of mitoses helps in diagnosis, special stains and cytogenetics may be helpful in arriving at a final diagnosis. CEOTs with prominent clear cells must be diagnosed with caution, as many clear cell neoplasms are malignant and further investigations are needed to exclude clear cell malignancies such as CCOC and other carcinomas with a clear cell component (for example, of renal or salivary origin) [38]. It is unclear to what extent difficulties in distinguishing clear cell CEOTs from CCOC has contributed to the reported apparent increased aggressiveness of clear cell CEOT.

### Non-Calcified and Langerhans Cell-Rich Variants of CEOT

The non-calcified variant of CEOT is the least reported variant (Table 3). To date, eight intraosseous cases and two extraosseous cases of non-calcified CEOT have been reported [39, 40]. The absence of calcification in CEOT may be due to the relative immaturity of the lesion, as long-standing tumors tend to have more calcifications than young, underdeveloped ones [41]. In a study of 19 patients with CEOT by Azevedo et al., the age of patients at the time of diagnosis was linked to the amount of calcification; older patients showing more calcifications [42]. This variant of CEOT usually appears as a radiolucent area on radiographs that may be misdiagnosed as an odontogenic cyst.

Many of these cases contain Langerhans cells (LC), which are antigen-presenting immune cells that are normally found in oral epithelium but have also been described in conventional CEOT in small numbers. If abundant, LC-rich lesions are considered a variant of CEOT [43, 44]. They appear histologically as clear cells, which contain Birbeck granules, within the tumor’s conventional pattern of polyhedral sheets of epithelial cells and amyloid-like material. Five of the cases reported so far were without associated calcification, all of whom presented in patients of Asian origin [45]. However, a Langerhans cell–rich case with calcification has been reported in one black individual [46], challenging the concept that ‘all CEOTs with a Langerhans cell component are non-calcified variants’. Diagnosis of this variant is based on either electron microscopic examination of the LC structure or positive staining of LCs for S100 and CD1a [46]. The natural history of this variant is not well described.

Histological examination was important in all of the reported cases of non-calcified CEOT, in order to evaluate the presence of the classic features of epithelial sheets and amyloid-like material. In one reported case there was a “poorly differentiated non-calcified CEOT” [41]. Others contained Langerhans cells. Takata et al. reported a case with a histologic appearance consistent with “pattern four” in the Ai-Ru subtypes of conventional CEOT [44]. It was suggested by Kaushal et al. that the non-calcified variant of CEOT behaves more aggressively than calcified CEOTs [39]. However, this contrasted with suggestions made in previous studies that most non-calcified CEOTs contain Langerhans cells, which may indicate a less aggressive lesion. More research in non-calcified CEOT cases with and without LCs is required to address this issue. There has been recent discussion regarding the nature of these non-calcifying, Langerhans cell-rich lesions [47]. This issue will be explored further later.

### Cystic/Microcystic Variant

Recently, a number of reports of cystic and microcystic variants of CEOT have been published. The initial report was of a large cystic lesion in a 15 year-old male, in which the lining demonstrated CEOT features [26]. The lesion was enucleated. A number of similar cases have been reported [48–50], and subsequently, a microcystic variant has also been described [25]. In this lesion, a pseudo-glandular appearance was reported in association with otherwise rather conventional CEOT histology. The natural history of these lesions is not known, but there have been no reports of recurrences so far.

### Combined CEOT-Adenomatoid Odontogenic Tumor

Although it is not a variant of CEOT, Adenomatoid odontogenic tumor (AOT) is worth mentioning in this context, as some contain CEOT-like areas. AOT is a separate odontogenic tumor with its own distinctive histological features. In 1983 Damm et al. reported an AOT that contained CEOT-like features and named it ‘combined epithelial odontogenic tumor’ [18]. Philipsen and Reichart reported 24 AOTs with some areas of CEOT-like components [23]. None of these combined AOTs /CEOT were dominated by CEOT-like areas. According to Ng and Siar, the behavior of these forms of AOT was no different from that of the conventional AOT and suggested they were benign hamartomas without any evidence of CEOT-like aggressive behavior, and none recurred [51]. Thus, combined CEOT-AOTs should be managed as conventional AOTs.

The designation of these cases as variants of CEOT has resulted in a dramatic widening of the histological spectrum of appearances that fall under the diagnostic umbrella of CEOT, far beyond the original histological description [1]. Furthermore, there are some odontogenic tumors that do not fit very well into the diagnostic criteria of the existing classification. This includes a number of lesions containing dentinoid and dispersed nests of tumor cells within a hyalinized stroma, which can share some histological features of CEOT. This raises an important issue as to the usefulness of tumor sub-classifications that develop incrementally, without periodic review of the variations in histological appearances in other tumors and integration of new insights from other molecular features including genomic analyses. It also raises questions regarding the usefulness of historical surveys of variants of this tumor, as, given progress in knowledge of the biology of odontogenic tumors, some variants which have been labelled as part of the CEOT family, may not be so.

In the present report, 26 sequentially accessioned cases from a single Oral and Maxillofacial Pathology Diagnostic Service from 1975 to 2017 have been analyzed. In these cases, diverse histomorphology was seen, but the index diagnosis was of a CEOT, or CEOT was included in the differential diagnosis. The whole cohort has been reviewed taking into account a number of other entities which have been described since the original diagnoses were made, particularly those in the early years of the cohort. In one case the resection specimen showed an odontogenic malignancy, with necrosis, a high mitotic rate and areas of de-differentiation. We excluded this as there was limited evidence of CEOT in the biopsy or resection. However, this does raise the issue of malignant CEOT, which we did not identify in the review of our diagnostic archive. A small number of individual case reports have been published, most of which show areas of conventional CEOT with associated malignant transformation [16, 52]. A detailed discussion of diagnostic features is beyond the scope of this review, however, as with ameloblastic carcinoma, this is fraught with difficulty. A combination of the use of a proliferation marker, such as Ki67, with histological features of malignancy may be useful, but this has not been assessed in a cohort of these lesions.

In our cohort, the “classic” appearance, as described in the initial Pindborg paper [1], was found in only 13/26 cases (50%). In our series, we defined this as a tumor demonstrating the described epithelial features (polyhedral cells with clear boundaries), and containing amyloid, in keeping with the WHO 2017 classification [28]. Other features, such as calcification and nuclear pleomorphism were variably present. Tumors with these histological features present little difficulty in diagnosis. Two other tumors were diagnosed as clear cell CEOT as, although they were dominated by a clear cell population, they also contained areas of “classic” CEOT, with amyloid.

The main differential diagnosis to be considered in the tumors with a significant clear cell component is Clear Cell Odontogenic Carcinoma (CCOC). CCOC is an intraosseous malignant neoplasm consisting of sheets, nests and cords of polygonal to round clear cells, usually separated by fibrous septa and often showing peripheral palisading [53]. The lesional clear cells are usually PAS positive, diastase sensitive and negative for mucicarmine (mucin). Congo Red (amyloid) is also negative. Histologically, CC-CEOTs that contain few epithelial islands with clear cells in an eosinophilic homogenous stroma need careful investigations in order to confirm them as CEOT. It is mandatory to identify the presence of amyloid for confirmation. Metastatic tumors that contain clear cells are most likely renal cell carcinoma, clear cell breast carcinoma or thyroid carcinoma and, therefore, immunomarkers such as RCC, CD10, PAX8, ER/PR, TTF-1 are useful [54].

In difficult cases or small biopsies, fluorescence in situ hybridization (FISH) for *EWSR1* gene rearrangement can be used to resolve this dilemma. *EWSR1* gene rearrangement is absent in CEOT, clearly separating CC-CEOT from CCOC. Bilodeau et al. analyzed 12 CCCa and 8 CCOCs for *EWSR-ATF1* FISH with 92% and 63% positive respectively. Subsequent Congo Red staining revealed that two of the CCOC that were negative for *EWSR1* rearrangement contained amyloid; therefore these were more likely to be hypocellular CEOTs rather than CCOC with hyalinized stroma [55]. A key element in this analysis is the availability of tissue which has not been decalcified. Unfortunately, a combination of unavailability of FFPE blocks, very old tissue and a high frequency of decalcification in our cohort meant that EWRSR1 rearrangement studies were either not possible, or failed, in our cohort.

In cases where a differential diagnosis was agreed after review, four included odontogenic fibroma (OdF) and sclerosing odontogenic carcinoma as differential diagnoses. On H&E, these cases resemble “pattern 4” in the subtypes described by Ai-Ru et al. [27], with dominance of a fibrous stroma component. The difficulties in distinguishing these entities have been recently discussed in the literature and are very relevant to addressing the issues of the uncertain nature of the non-calcifying CEOT variants. As highlighted recently by Ide et al. [47], differential diagnosis of odontogenic fibroma (OdF) has been raised in these lesions and, indeed, there is much to suggest (including a lack of recurrence) that they may represent odontogenic fibromas, rather than non-calcifying CEOTs. This is reinforced in the case series reported by Eversole [56], where a small number of the 65 OdFs described contained both ODAM positive amyloid and Langerhans cells. It is worth noting that this issue was raised in the 1971 WHO classification, in relation to the differential diagnosis of non-calcifying CEOT and cellular OdF [57].

We considered sclerosing odontogenic carcinoma as a differential diagnosis in some cases (Table 1). This tumor has now been added to the WHO classification [28], but is somewhat controversial, and clear diagnostic criteria have not been established. Perineural invasion was not seen in any of these cases where this was considered as a diagnosis.

Three of these cases contained dentinoid. The significance of this is unclear, but in two cases, we included odontogenic carcinoma with dentinoid in the differential diagnosis, as these tumors presented some features similar to the case reports of this entity [58]. In particular, this was considered in cases where the original diagnosis was rather uncertain, where CEOT was a suggested diagnosis whilst acknowledging the tumor was difficult to classify. This indicates that the classification, and what may be considered to fall within the diagnostic remit of CEOT, may further evolve as other odontogenic entities are described and their diagnostic criteria established.

## Conclusion

The development of diagnostic criteria for a tumor is an iterative process and the description and acceptance of tumor variants is limited to some degree by the lack of appropriate molecular tools to confirm or refute the placing of a particular tumor into its place on the classification. The description of a number of the variants of CEOT very much falls into this trap. Whilst some of the variants are most likely true variants of CEOT, it is becoming increasingly apparent that others are most likely a part of the spectrum of other odontogenic entities. This includes CCOC (now with *EWSR1* cytogenetics to aid diagnosis) and odontogenic fibroma. Further refinement will most likely require a collaborative international approach to collect sufficiently large cohorts of these cases allow a more comprehensive molecular characterization of this group of lesions. In this way, more variants may be defined as other entities, whilst the true spectrum of CEOT is established. Such analysis may also aid in defining the histogenesis of these lesions.

This will not be without its challenges: many of the cases of CEOT are decalcified, which may significantly compromise the quality of genomic information which can be obtained from these specimens. To this end, careful consideration will have to be given to a concerted international effort to collect samples which have been optimally collected, stored and processed. The development of an international prospective database, with associated availability of both fixed and fresh material, which has not undergone harsh decalcification will be needed, and this could be coordinated via various international specialist societies. This will then allow for a program of translational research, which can include multi-omics analyses of these tumors.
